# Fracture resistance among post and core systems: An *in vitro* study

**DOI:** 10.6026/973206300221986

**Published:** 2026-04-30

**Authors:** Kamil Shahnawaz, Swati Sagar, Kartik Salarias, Ankita Grace Vijay, R Vignesh, Avantika Vijaysingh Jadhav

**Affiliations:** 1 Department of Dentistry, Darbhanga Medical College & Hospital, Laheriasarai, Darbhanga, Bihar-846003, India; 2 Department of Conservative Dentistry & Endodontics, Himachal Dental College, Sundernagar, Himachal Pradesh-175019, India; 3 Department of Conservative Dentistry and Endodontics, Sri Aurobindo College of Dentistry, Sri Aurobindo University, Indore, Madhya Pradesh-452001, India; 4 Department of Conservative Dentistry & Endodontics, Sree Balaji Dental College & Hospital, Pallikaranai, Chennai-600100, Tamil Nadu, India; 5 Department of Prosthodontics, Bharati Vidyapeeth Dental College and Hospital, Sector 7, CBD Belapur Navi Mumbai, Maharashtra, India

**Keywords:** Fracture resistance, endodontically treated teeth, post-and-core systems, fiber posts, cast metal posts, prefabricated metal posts

## Abstract

Endodontically treated teeth are highly susceptible to fracture due to loss of structural integrity. Therefore, it is of interest to compared fracture resistance and failure patterns among cast metal, prefabricated metal and fiber post-and-core systems under standardized conditions. Thirty mandibular premolars were endodontically treated, restored and subjected to oblique compressive loading at 135°C until failure. Fiber posts showed the highest fracture resistance (615 ± 50 N) and predominantly favorable, repairable fractures (80%), whereas metal posts exhibited lower resistance and mainly unfavorable root fractures (p < 0.001). Thus, we show controlled biomechanical evidence supporting fiber posts as a superior restorative option for preserving root integrity.

## Background:

Endodontically treated teeth exhibit reduced fracture resistance due to loss of coronal structure and alteration of dentin biomechanics [1]. Removal of tooth structure during access preparation and post space preparation further compromises structural integrity [2]. Restoration of such teeth therefore requires reinforcement in addition to retention. Post-and-core systems are widely used when insufficient coronal tooth structure remains to retain a definitive restoration [3]. The mechanical behavior of a restored tooth depends largely on the elastic modulus and design of the post material [4]. Cast and prefabricated metal posts possess high stiffness, which may concentrate stresses within the radicular dentin under functional loading. This stress concentration increases the risk of vertical root fractures, which are often non-repairable [5]. In contrast, fiber-reinforced composite posts demonstrate an elastic modulus closer to dentin, allowing more uniform stress distribution [6]. Contemporary evidence suggests that fiber posts may improve fracture patterns and enhance reparability, although reported differences in fracture resistance remain inconsistent across studies [7]. Biomechanical outcomes are also influenced by post length, diameter, ferrule height, luting cement and loading direction [8]. Standardization of these variables is essential to obtain reliable comparative data [9]. Many previous investigations demonstrate methodological heterogeneity, limiting direct comparison of post systems. Furthermore, failure mode and repairability are clinically more relevant than fracture load alone [10]. Therefore, it is of importance to compare the fracture resistance and failure patterns of teeth restored with cast metal, prefabricated metal and fiber post- and-core systems in this study.

## Materials and Methods:

This *in vitro* experimental study was conducted using thirty extracted human mandibular premolars of similar dimensions. Teeth were free from caries, cracks, restorations and resorption. Specimens were cleaned and stored in distilled water until use. All teeth underwent standardized endodontic treatment using rotary nickel-titanium instrumentation. Canals were obturated with gutta-percha and resin sealer using lateral condensation. Crowns were sectioned at the cementoenamel junction to obtain a uniform root length of 14 mm. Specimens were randomly divided into three groups (n=10) according to the post-and-core system. Group I received custom cast metal post- and-core restorations fabricated in nickel-chromium alloy. Group II received prefabricated stainless-steel posts cemented with resin cement followed by composite core build-up. Group III received glass fiber posts cemented with dual-cure resin cement and composite core build-up. Post space was prepared to 9 mm while maintaining a 5 mm apical seal. Core height was standardized at 5 mm in all groups. Specimens were embedded vertically in acrylic resin blocks, leaving 2 mm of coronal root structure exposed to simulate a ferrule effect. Each specimen was subjected to static compressive loading at 135°C to the long axis using a universal testing machine at 1 mm/min crosshead speed. The maximum fracture load was recorded in Newton's. Fracture mode was categorized as favorable or unfavorable based on restorability. Data were analyzed using SPSS version 26.0. Mean fracture resistance values were compared using one-way ANOVA followed by Tukey's post hoc test. Fracture modes were analyzed using the chi-square test. Statistical significance was set at p < 0.05.

## Results:

All thirty mandibular premolars were successfully endodontically treated and restored without procedural complications. The mean root length of specimens was 14.2 ± 0.5 mm, ensuring dimensional homogeneity. Post space length, apical seal and core height were standardized at 9 mm, 5 mm and 5 mm, respectively, across all groups. The mean fracture resistance differed significantly among the three post systems (F = 17.42, p < 0.001). Fiber posts exhibited the highest mean fracture resistance (615 ± 50 N), followed by cast metal posts (520 ± 42 N) and prefabricated metal posts (475 ± 38 N). The minimum and maximum fracture values ranged from 540- 690 N for fiber posts, 460-590 N for cast posts and 420-540 N for prefabricated posts. Post hoc Tukey analysis showed significant differences between fiber and cast posts (p = 0.002) and between fiber and prefabricated posts (p < 0.001), while no significant difference was observed between the two metal groups (p = 0.081). Favorable fractures occurred in 80% of fiber specimens compared with 30% in cast posts and 40% in prefabricated posts. Root fractures predominated in metal groups, occurring in 80% of cast and 70% of prefabricated specimens, whereas 70% of fiber post failures were coronal. Repairable fractures were recorded in 80% of fiber posts compared with 20% in cast and 30% in prefabricated groups. Correlation analysis demonstrated significant positive associations between fracture resistance and post length (r = 0.45, p = 0.018) and core height (r = 0.39, p = 0.042), while post diameter was not significantly correlated (p = 0.127). Regression analysis identified post type (β = 86, p = 0.001), post length (β= 12, p = 0.024) and core height (β= 15, p = 0.019) as significant predictors of fracture resistance. Table 1 (see PDF) shows that all thirty specimens were mandibular premolars with a mean root length of 14.2 ± 0.5 mm and a mean donor age of 42.6 ± 7.4 years. Table 2 (see PDF) demonstrates uniform post-space preparation across groups with identical post length (9.0 ± 0.2 mm), apical seal (5.0 ± 0.0 mm) and core height (5.0 ± 0.0 mm). Table 3 (see PDF) compares mean fracture resistance and shows the highest value in the fiber post group (615 ± 50 N), followed by cast metal posts (520 ± 42 N) and prefabricated metal posts (475 ± 38 N). Table 4 (see PDF) indicates higher median fracture resistance in the fiber group (610 N) compared with cast (518 N) and prefabricated posts (474 N), with corresponding 95% confidence intervals of 585-645 N, 495-545 N and 451-499 N. Table 5 (see PDF) demonstrates a statistically significant difference in fracture resistance among groups (F = 17.42, p < 0.001). Table 6 (see PDF) compares pairwise differences and shows significant superiority of fiber posts over cast posts (mean difference -95 N, p = 0.002) and prefabricated posts (mean difference -140 N, p < 0.001), while cast and prefabricated posts do not differ significantly (p = 0.081). Table 7 (see PDF) depicts favorable fractures in 80% of fiber posts compared with 30% in cast and 40% in prefabricated groups. Table 8 (see PDF) highlights that root fractures occurred in 80% of cast and 70% of prefabricated specimens, whereas 70% of fiber post failures were coronal. Table 9 (see PDF) indicates significant positive correlations between fracture resistance and post length (r = 0.45, p = 0.018) and core height (r = 0.39, p = 0.042), while post diameter shows no significant correlation (p = 0.127). Table 10 (see PDF) demonstrates that post type (β= 86, p = 0.001), post length (β= 12, p = 0.024) and core height (β= 15, p = 0.019) are significant predictors of fracture resistance in regression analysis. Table 11 (see PDF) shows repairable fractures in 80% of fiber posts compared with 20% in cast and 30% in prefabricated groups. Table 12 (see PDF) compares overall outcomes and shows the highest fracture resistance, greatest percentage of favorable fractures (80%) and highest repairability (80%) in the fiber post group.

## Discussion:

This study compared fracture resistance and failure patterns of cast metal, prefabricated metal and fiber post systems under standardized conditions. Fiber posts demonstrated significantly higher fracture resistance than both metallic systems. Fiber posts also produced predominantly favorable and repairable fractures. Metal posts were associated mainly with unfavorable root fractures. The superior performance of fiber posts is biomechanically plausible. Their elastic modulus approximates dentin and allows more uniform stress distribution under oblique loading [11]. Recent investigations consistently report reduced stress concentration within radicular dentin when fiber posts are used. In contrast, metallic posts exhibit high stiffness and transmit concentrated stresses to cervical and apical regions. This stress concentration predisposes the root to vertical fracture. Although some contemporary studies show comparable ultimate fracture loads when adequate ferrule is present, failure pattern remains a decisive clinical variable. Repairable coronal fractures preserve retreatment options and improve prognosis [12]. In the present study, 80% of fiber post failures were repairable. Only 20-30% of metal post failures were repairable. This difference is clinically significant even when absolute fracture loads overlap. Regression analysis further demonstrated that post type, post length and core height independently influence fracture resistance. These findings reinforce current evidence that structural design parameters interact with material properties [13]. Adequate post length improved fracture resistance without compromising the apical seal. Core height also showed a positive association with resistance values. Thus, restorative geometry must be optimized alongside material selection. By controlling post length, apical seal, ferrule simulation and loading angle, this study minimizes methodological variability reported in earlier literature [14]. The integration of fracture load, fracture mode, repairability, correlation and regression modeling provides a multidimensional biomechanical evaluation. This approach clarifies the relative contribution of material type and structural parameters in determining failure behavior [15]. This investigation remains limited by its *in vitro* design and absence of cyclic fatigue or thermal aging. Static loading does not replicate long-term intraoral conditions. Clinical extrapolation must therefore be cautious. Future research should incorporate fatigue simulation and long-term clinical follow-up to validate structural performance. Overall, fiber posts demonstrated superior biomechanical behavior and more favorable failure characteristics compared with metallic systems under standardized testing conditions.

## Conclusion:

Fiber post systems demonstrated significantly higher fracture resistance and predominantly repairable fracture patterns compared with metallic posts under standardized testing conditions. Post type, post length and core height significantly influenced structural performance. Fiber posts should be preferred when preservation of root integrity and long-term restorability are primary clinical objectives.
